# Childhood adversity and depression of older adults: the moderating effect of social participation

**DOI:** 10.3389/fpsyg.2024.1376155

**Published:** 2024-08-01

**Authors:** Ping Wang, Xin Cheng, Nan Zhang, Huilin Liu

**Affiliations:** ^1^School of Management, Xi’an University of Science and Technology, Xian, China; ^2^Department of Rehabilitation, Xijing Hospital, Air Force Medical University, Xian, China

**Keywords:** depression, childhood adversity, social participation, regulatory effect, older adults

## Abstract

**Objective:**

Examine the effect of childhood adversity on depression in older adults and the regulatory impact that social participation has on depression.

**Methods:**

Based on 6,704 standard-compliant research subjects, single factor analysis, multiple linear regression model, and tendency score matching were used to analyze the impact of childhood adversity on depression in older adults and the regulatory effect of social participation.

**Results:**

The depression rate is higher among women, young age, low education, unmarried, in agricultural households, older adults with low annual income, pre-retirement work type in agriculture, non-drinking, and those with two or more chronic diseases (*p* < 0.05). Children who experienced adversity as children are more likely to suffer from depression as adults (*β* = 0.513, 0.590, 0.954, 0.983, 1.221, 0.953, 0.718; *p* < 0.05). Through the tendency score, the result is matched with the endogenous test. As well, older adults are more likely to suffer psychological damage from a greater number of childhood adversities in their early years (*β* = 1.440, 2.646, 4.122; *p* < 0.001). It has been shown that social participation will reduce the negative impact of low-income family economic circumstances on depression among older adults of all ages (*β* = −0.459,-0.567; *p* < 0.01), aggravate depression resulting from “neighborhood void of mutual assistance” and “no more fun to play” for older adults of all ages (*β* = 1.024, 0.894; *p* < 0.01), and exacerbate depression resulting from “loneliness because there are no friends” for the oldest old (*β* = 0.476, 0.779; *p* < 0.05).

**Conclusion:**

Older adults who experience childhood adversity are more likely to suffer from depression. Social participation plays a regulatory role in the relationship between childhood adversity and depression in older adults. For older adults’ mental health to improve, family and social adversity should be prevented, and moderate participation in society should be encouraged.

## 1 Introduction

As the aging population deepens, psychological health issues among older adults are increasingly highlighted in the process of social development, drawing growing attention from academia and society alike (Liu et al., 2021). Depression is a prevalent and chronic psychological issue among elderly populations, characterized by persistent emotional downturns (Li and Luo, 2023). It is associated with high rates of disability and can severely impact quality of life, potentially leading to suicide (Li et al., 2014; Kim and Lee, 2019). In the context of rapid aging populations, recent studies have shown a depression prevalence rate as high as 20.0% among older adults in China (Tang et al., 2021), underscoring the urgent need for attention to the mental health of older adults. Numerous studies have explored predictive factors for depression in older adults, including age, gender, retirement status, and income (Zunzunegui et al., 2007; Yunming et al., 2012). Scholars increasingly examine individual health from a life-course perspective (Warren, 2016), recognizing childhood experiences as impactful across the lifespan, particularly influencing late-life health outcomes (Angelini et al., 2019).

Childhood adversity refers to intense stressors experienced by individuals during childhood (Bellis et al., 2019), characterized by childhood abuse and dysfunctional family environments. From the perspective of life course theory, childhood adversity not only causes immediate harm to individuals but also exerts cumulative effects that persist throughout the entire lifespan, influencing both psychological health. These early experiences have profound impacts on social, emotional, and cognitive development, ultimately manifesting as an increased risk of depression in old age (Yang et al., 2020). Several studies have shown that people with a history of childhood adversity are more likely to suffer from depression (Zhao et al., 2022), indicating that childhood adversity is an important socio-environmental factor in depression (Grummitt et al., 2021). It is worthwhile to note, however, that not all older adults who experience adversity suffer from depression. On the contrary, many may even adapt to the difficult conditions (Ye and Zhang, 2021). Studies have found that protective factors, such as genetics and resilience, may moderate the relationship between childhood adversity and depression (Beutel et al., 2017; Ashy et al., 2020; Yu et al., 2021). In addition, the presence of social support has been shown to mitigate the adverse impact of childhood adversity on late-life depression (Zhao et al., 2019).

Social participation is an essential component of successful aging strategies (Choi et al., 2021), defined as engagement in social activities such as maintaining social relationships, participating in community events, or volunteering (Piškur et al., 2014). It contributes to enhancing overall quality of life in later years. According to a study based on an elderly population in Switzerland, rich and diverse social participation increases life satisfaction (Baeriswyl and Oris, 2023). Older adults who are more socially engaged feel less lonely and dissatisfied, positively affecting their physical and mental health. Multiple studies have shown that social participation helps older adults gain social support, exercise their bodies, and delay the decline of cognitive, cardiovascular, and endocrine functions. These activities significantly prevent and alleviate depression in older adults (Mackenzie and Abdulrazaq, 2021; Donoso et al., 2023), and improve their mental health. Moreover, social participation can alleviate depression and other negative emotions caused by the loss of social roles in older adults, fostering a renewed sense of self-worth and maintaining vitality and positivity in their lives (Knapp, 1977). Although existing research has demonstrated the impact of adverse childhood experiences on depression in older adults, there is a lack of studies exploring whether social participation moderates this relationship, especially within the Chinese older adults. Therefore, based on the 2014 “Life Course Survey of Middle-aged and Elderly People in China” and the 2018 China Health and Retirement Longitudinal Study data from 28 provinces, this study evaluated the relationship between childhood adversity and depression in 6,704 older adults aged 60 and above. Additionally, it examined the moderating effect of social participation on this relationship to develop effective early intervention measures for depression.

## 2 Materials and methods

### 2.1 Object of study

This study uses data from the China Health and Retirement Longitudinal Survey (CHARLS), a large-scale interdisciplinary tracking project carried out jointly by the China Social Science Survey Center at Peking University and the Peking University Youth League Committee. As part of CHARLS, a national baseline survey was conducted in 2011–2012, followed by three national follow-up surveys in 2013, 2015, 2018 and 2020, and a life course survey in 2014. It was conducted using the Probability Proportionate to Size (PPS) sampling method on 150 counties and 450 communities in 28 provinces, making the sample nationally representative. Using the particular survey data from the “China Middle-aged and Elderly Life Course Survey” in 2014 and 2018, this study horizontally merges the two databases by matching the elderly identity code variables. Childhood adversity came from the 2014 Chinese Middle-aged and Elderly Life Course Survey, while depression, social participation and control variables came from the 2018 China Health and Elderly Care Tracking Survey. In this study, childhood adversity is a key variable, so we excluded samples that did not participate in the 2014 special survey and those aged 45–59, resulting in a reduction of 11,751 samples. Subsequently, we excluded 1,297 samples with missing or abnormal values for the three main variables: childhood adversity, depression, and social participation. Ultimately, we obtained a final sample of 6,704 older adults aged 60 and above.

### 2.2 Description of variables

#### 2.2.1 Dependent variables

A 10-item brief table of the Center for Epidemiological Research Depression Scale (CES-D10) was used to assess depression tendency in the sample. Globally, the Center for Depression Studies Depression Scale (CES-D) and the CES-D10 short form are widely used to screen depression symptoms in the general population (Radloff, 1977). There are 10 items on the CES-D10 scale, with 8 reflecting negative emotional experiences (being annoyed, having difficulty concentrating, feeling low, struggling to do things, being afraid, not sleeping well, being lonely) and 2 reflecting positive emotional experiences (being hopeful about the future, being happy). 0 = rarely, 1 = not much, 2 = sometimes, and 3 = often. The two options with positive tendencies are reverse-coded, and the total score ranges from 0 to 30. In general, the higher the scale value, the worse the mental health status and the higher the level of depressive tendencies. The Cronbach’s alpha coefficient of the scale is 0.83, indicating good internal consistency.

#### 2.2.2 Independent variables

According to the previous study (Liu and Li, 2022), a total of seven childhood adversity, including family and social environment dimensions, were included: low-income family economic situation, two weeks or more of depression in the caregiver, the caregiver with severe physical disabilities, poor relationships with parents, lack of mutual assistance within the neighborhood, lack of good playmates, loneliness due to lack of friends. Older adults who have experienced this childhood adversity will be coded as 1, and vice versa; they will be coded as 0. At the same time, this paper will test the “dose–response” effect of childhood adversity. When an interviewee experiences one childhood adversity, score 1 point, ranging from 0 to 7 points (Wicks et al., 2005; Hu, 2021). To examine the dose–response effect of childhood adversity, this study categorized the respondents based on the distribution of “number of childhood adversities,” using 0 as the reference group, and transformed the “number of childhood adversities” into three dummy variables.

#### 2.2.3 Control variables

Four types of control variables were analyzed in this study, including demographic characteristics, economic status, behavioral characteristics, and health status. In particular, they include gender (male, female), age, type of household (agricultural, urban), education level (illiterate, primary school, junior high school and above), marital status (non-married, married), annual income number of older adults (<6.87; 6.87 ~ 9.49;>9.49), type of work before retirement (agriculture, government departments/ institution/non-profit organization, business/individual), alcohol consumption (no, yes), chronic disease (no chronic disease, one chronic disease, two or more chronic diseases).

#### 2.2.4 Moderating variables

The social participation of older adults served as a moderating variable in this study. According to the CHARLS questionnaire, respondents were asked if they had participated in 11 social activities during the past month, such as visiting the door, socializing with friends, playing mahjong, chess, cards, and going to the community room. Each activity was assigned a value of “0” if the response was “not participating” and “1” if the response was “participating” (Liao et al., 2022). The total number of social activities participated in by older adults was summed to obtain an index ranging from 0 to 11, which was used to measure social participation.

### 2.3 Methods

The data were analyzed using STATA15.1 software, and single-factor analysis was used to compare the depression degrees of older adults based on their essential characteristics. An analysis of childhood adversity and depression in older adults was conducted using multiple linear regression models. The robustness of the relationship between childhood adversity and depression was tested in order to alleviate endogenous problems. To analyze the “dose–response” effects of 7 childhood adversity items, the “number of childhood adversity experiences” was transformed into a dummy variable and analyzed with a multiple linear regression model. Furthermore, the moderating effect was examined by combining the product term of social participation with the seven items of childhood adversity.

## 3 Results

### 3.1 Basic situation

A total of 6,704 older adults were surveyed in this study, of which 3,325 (49.60%) were males and 3,379 (50.4%) were females; their ages ranged from 60 to 118, with an average age of 70.24 years old; 5,222 (77.89%) belonged to agricultural households and 1,482 (22.11%) were from urban households; 2,053 (30.62%) were illiterate and 2,993 (44.64%) in primary school, 1,658 (24.73%) in junior high school and above; 2,355 (35.13%) not in marriage (including widowed, divorced and unmarried), 4,349 (64.87%) in marriage; 6,187 (92.29%) working in agriculture as type of work before retirement; 121(1.8%) working in government departments/institutions/non-profit organizations, 396 working in enterprises/individuals (5.91%); 2,051 (30.59%) drinking alcohol, 4,653 (69.41%) not drinking alcohol; 3,466 (51.70%) without chronic diseases, 1,925 (28.71%) with one chronic disease, 1,313 (19.59%) with two or more chronic diseases; 1,908 with annual income <6.84 ~ 9.49, and 1,668 with annual income >9.49. 2,217 (33.07%) had no childhood adversity, 1,977 (29.49%) had one childhood adversity, 1,343 (20.03%) had two childhood adversity, and 1,167 (17.41%) had three or more childhood adversity.

### 3.2 Univariate analysis of depression status in older adults with different characteristics

Based on univariate analysis of Table 1, there are significant differences in depression degree among older adults based on their gender, age, education level, marital status, type of household, income, type of work before retirement, alcohol consumption, chronic disease, and childhood adversity experience (*p* < 0.05). Female older adults were more likely to suffer from depression than male older adults; The young old had a higher depression degree than the oldest old. Those who are illiterate have the highest degree of depression, while those who have a high school education or more have the lowest. The unmarried were more depressed than the married. There is a higher depression degree among older adults in agricultural households than among older adults in urban households. There was a greater prevalence of depression among those who worked in agriculture before retirement. Those who did not drink alcohol were more depressed than those who drank alcohol. Depression rates were highest among older adults with two or more chronic diseases and lowest among older adults without chronic diseases. The depression level of older adults who had faced “the caregiver with severe physical disabilities” was the highest, and the depression level of older adults who had faced a “low-income family economic situation” was the lowest. Table 1 shows the results.

**Table 1:** Comparison of depression in older adults with different characteristics.

Variant		Depression (*x* ± *s*)	*F*	*P*
Gender	Female	20.49 ± 11.37	74.611	<0.001
	Male	18.29 ± 9.40		
Age	60 ~ 69	19.78 ± 9.13	29.422	<0.001
	≥70	18.94 ± 11.90		
Education level	Illiterate	20.98 ± 13.14	64.962	<0.001
	Secondary schools	19.58 ± 9.60		
	Junior high school and above	17.09 ± 7.53		
Marital status	Unmarried	19.42 ± 8.89	22.031	0.020
	Married	19.35 ± 12.95		
Type of household	Agriculture	20.06 ± 10.87	32.039	<0.001
	Cities and towns	17.08 ± 8.67		
Annual income number of older adults	<6.87	21.06 ± 11.67	84.496	<0.001
	6.87 ~ 9.49	19.83 ± 10.54		
	>9.49	16.68 ± 8.24		
Type of work before retirement	Agriculture	19.54 ± 10.63	8.607	<0.001
Government departments /institutions / nonprofit organizations		16.74 ± 7.09		
Businesses/ self-employed entrepreneur		17.88 ± 8.84		
Alcohol consumption	No	19.93 ± 11.10	39.884	<0.001
	Yes	18.18 ± 8.86		
Chronic disease status	0 = no chronic disease	18.79 ± 10.13	4.738	<0.001
1 = a chronic disease		19.66 ± 10.34		
2 = two or more chronic diseases		20.62 ± 11.50		
Lack of mutual assistance within the neighborhood	No	19.08 ± 10.25	41.811	<0.001
	Yes	21.54 ± 11.80		
Lack of good playmates	No	18.82 ± 10.18	62.471	<0.001
	Yes	21.15 ± 11.22		
Lonely due to lack of friends	No	19.06 ± 10.35	47.673	<0.001
	Yes	21.71 ± 11.19		
Poor family economic situation	No	18.68 ± 10.37	52.620	<0.001
	Yes	20.59 ± 10.60		
Two weeks or more of depression in the caregiver	No	18.93 ± 10.54	62.448	<0.001
	Yes	21.58 ± 9.99		
The caregiver with severe physical disabilities	No	19.29 ± 10.50	15.401	<0.001
	Yes	21.72 ± 10.19		
Poor relationship with parents	No	19.01 ± 10.50	35.415	<0.001
	Yes	20.88 ± 10.33		

### 3.3 Analysis of the influence of childhood adversity on depression in older adults

Multiple linear regression was conducted using depression score as the dependent variable and seven childhood adversities as the independent variables. Model 1 showed that after considering childhood adversities, lack of mutual assistance within the neighborhood, lonely due to lack of friends, two weeks or more of depression in the caregiver, the caregiver with severe physical disabilities, and having a poor relationship with parents, older adults were more likely to be depressed (*p* < 0.01). Adding moderating and controlling variables to model 1, the results show that childhood adversity’s coefficients have all declined but still have strong explanatory power for depression among older adults. Among older adults, social participation, age, education, Logarithm of the annual income of older adults, alcohol consumption, urban household registration, marriage, and work type before retirement are non-agricultural (working for government departments, institutions, non-profit organizations, or individuals) decreased depression risk (*p* < 0.05). The higher number of chronic diseases would enhance the tendency to depression in older adults (*p* < 0.05). Table 2 shows the results.

**Table 2:** Analysis of the influence of childhood adversity on depression of older adults.

Variant	Model1		Model2	
	*β*	SD	*β*	SD
Social environment				
Lack of mutual assistance within the neighborhood	0.968^***^	0.239	0.513^**^	0.232
Lack of good playmates	1.026^***^	0.186	0.590^***^	0.182
Lonely due to lack of friends	0.973^***^	0.243	0.954^***^	0.233
Family environment				
Poor family economic situation	1.101^***^	0.166	0.983^***^	0.160
Two weeks or more of depression in the caregiver	1.470^***^	0.211	1.221^***^	0.203
The caregiver with severe physical disabilities	1.023^***^	0.379	0.953^***^	0.363
Poor relationship with parents	0.755^***^	0.193	0.718^***^	0.185
Social participation			−0.517^***^	0.077
Gender (Female = 0)			−1.490^***^	0.175
Age			−0.089^***^	0.011
Education level (Illiterate = 0)			−0.473^***^	0.123
Marital status (Not married =0)			−0.386^**^	0.174
Type of household (Agriculture = 0)			−0.793^***^	0.209
Annual income number of older adults			−0.169^***^	0.038
Type of work before retirement (Agriculture = 0)			−0.642^***^	0.160
Alcohol consumption (No = 0)			−0.488^***^	0.177
Chronic disease status (No chronic disease = 0)			0.821^***^	0.069
Constant	7.622^***^	0.113	16.970^***^	0.895
*F*	44.620		56.450	
*R* ^2^	0.045		0.125	
Sample size	6,704		6,704	

* * *, * *, and * are significant at the statistical level of 1, 5 and 10% respectively.

### 3.4 Propensity score matching

According to the model above, the effect of childhood adversity on depression in older adults may be endogenous since older adults with a higher propensity to be depressed are perhaps more likely to report experiencing more childhood adversity, causing selectivity bias. Propensity score matching was used in the present study to mitigate the endogeneity problem caused by selectivity bias, including nearest neighbor, radius, and kernel matching. According to three matching methods, Table 3 shows the average treatment effects on the treated (ATT) of the seven childhood adversities on depression in older adults. All T-values are significant at 1%. The similarity of the results obtained by different matching methods suggests that ATT estimates are robust, suggesting that childhood adversities are still relevant to depression.

**Table 3:** Matching analysis results of tendency score.

Variables		Nearest neighbor matching		Radius match		Nuclear matching	
		ATT	SD	ATT	SD	ATT	SD
Social environment	Lack of mutual assistance within the neighborhood	1.519^**^	0.626	0.911^**^	0.444	0.898^***^	0.444
	Lack of good playmates	0.962^**^	0.474	1.213^***^	0.334	1.208^***^	0.335
	Lonely due to lack of friends	1.202^**^	0.586	1.347^***^	0.428	1.285^***^	0.428
Family environment	Poor family economic situation	1.211^***^	0.380	0.882^***^	0.289	0.885^***^	0.289
	Two weeks or more of depression in the caregiver	1.031^**^	0.490	1.362^***^	0.344	1.332^***^	0.344
	The caregiver with severe physical disabilities	2.240^**^	0.885	1.612^***^	0.608	1.583^***^	0.608
	Poor relationship with parents	1.047^**^	0.420	1.237^***^	0.317	1.227^***^	0.317

* * *, * *, and * are significant at the statistical level of 1, 5 and 10% respectively.

### 3.5 “Dose–response” effect

The “dose–response” effect of childhood adversity was tested with a multiple linear regression model using a dummy variable representing the number of childhood adversity experienced by the sample as the independent variable and depression score as the dependent variable. Table 4 showed that childhood adversity had a significant dose–response effect on depression in older adults. Older adults with one childhood adversity scored higher on depression by 1.440 units; Older adults with two childhood adversities scored higher on depression by 2.646 units; And older adults with three childhood adversities scored higher on depression by 4.122 units. Specifically, the more childhood adversity an older adult experiences, the more significant the impact on their depression levels.

**Table 4:** “Dose–response” effect.

Variables	Depression			
	Base model		Full model	
	*β*	SD	*β*	SD
Experience a childhood adversity	1.698^***^	0.317	1.440^***^	0.314
Experience two childhood adversities	3.245^***^	0.354	2.646^***^	0.355
Experience three or more childhood adversities	5.081^***^	0.371	4.122^***^	0.376
Constant	28.126^***^	1.190	33.494^***^	1.471
Sample size	6,704		6,704	

* * *, * *, and * are significant at the statistical level of 1, 5 and 10% respectively.

### 3.6 Adjustment test

Using the total number of social participation activities in the sample of 6,704 as the moderating variable, we examined the moderating effect of social participation on childhood adversity and depression. To demonstrate the differences in moderating effects for different age groups of older adults, the sample was divided into two groups: the young old and the oldest old. Results showed that among older adults of all ages, the product terms “poor family economic situation” and “social participation” negatively correlated with depression (*p* < 0.05). Older adults of all ages were positively associated with “lack of mutual assistance within the neighborhood” and social participation, as well as the product term “lack of good playmates” and social participation. It was found that the product term “loneliness due to lack of friends” and social participation were positively associated with depression among the oldest old (*p* < 0.1), which is statistically significant. Table 5 shows the results.

**Table 5:** Analysis of the moderating effect of social participation on childhood adversity and depression of older adults.

Variables	Product term	The young old (60 ~ 70)		The oldest old (>70)	
		*β*	*SD*	*β*	SD
Social environment	Lack of mutual assistance within the neighborhood × social participation	1.024^**^	0.417	0.894^**^	0.395
	Lack of good playmates×social participation	0.476^*^	0.283	0.779^**^	0.312
	Lonely due to lack of friends × Social participation	−0.478	0.312	0.736^*^	0.427
Family environment	Poor family economic situation × Social participation	−0.459^**^	0.211	−0.567^**^	0.274
	Two weeks or more of depression in the caregiver × Social participation	0.127	0.265	0.196	0.346
	The caregiver with severe physical disabilities × Social participation	0.123	0.523	0.777	0.750
	Poor relationship with parents × Social participation	−0.030	0.241	0.061	0.335

* * *, * *, and * are significant at the statistical level of 1, 5 and 10% respectively.

## 4 Discussion

### 4.1 Single factor analysis of depression in older adults with different characteristics

According to the study, female older adults are more likely to be depressed, which corresponds to traditional Chinese medicine’s theory that “women are more depressed” (Wang et al., 2022). Possibly, this is because women are by nature sensitive, and they have weaker emotional stability and the ability to resist pressure (Hu, 2021). It has been found that the oldest-old have a lower depression level. According to “social–emotional selection theory,” older adults are better at reconciling the conflict between behaviors and ideas due to years of accumulation and experience fewer negative emotions (Chen and Jordan, 2018). Unmarried older adults have a higher risk of depression, mainly because they face greater pressure from the public, which negatively affects their psychological well-being (Li, 2021). As a result of their low incomes, agricultural household registration, and farming type of work before retirement, older adults with agricultural household registration are more likely to suffer from depression since the above-mentioned old adults cannot obtain more social resources easily. There is a wide gap between urban and rural economic and healthcare resources, making it difficult for them to obtain relevant medical care and social support (Angelini et al., 2019; Yuan et al., 2023); A higher level of education leads to a lower level of depression among older adults because it allows them to understand their situation from the correct perspective of culture and economics. Meanwhile, education can also increase their employment opportunities, improve their incomes and socioeconomic status, and lower their depression levels (Back and Lee, 2011). The study indicates that older adults who do not drink alcohol have a higher degree of depression, which is consistent with existing research (Cai et al., 2022). It is mainly because drinking alcohol can help older adults reduce stress so that they can “drown their sorrows,” and moderate drinking can help them cope with depression. Thus, we need to focus on improving the mental health of those mentioned above key older adults by implementing targeted early intervention strategies.

### 4.2 Analysis of the influence of childhood adversity on depression in older adults

According to multiple linear regression results, older adults who experienced childhood adversity in a family or social setting are more depressed, which is consistent with existing studies (Domènech-Abella et al., 2021; Cross et al., 2023). A propensity score matching test revealed that childhood adversity still had an explanatory effect on depression in older adults despite some endogenous issues in the original model. Compared with older adults without childhood adversity, those who experienced childhood adversity were more likely to experience depression. This result is consistent with the findings reported in previous studies (Merrick et al., 2017). Furthermore, the dose–response results showed that childhood adversities had a more significant impact on depression levels in older people when the number of adversities was high. This implies that an increase in the number of childhood adversities exacerbates the negative impact on children’s psychological development, leading to higher outcome risks (Finkelhor et al., 2011). This conclusion highlights the cumulative effect of multiple childhood adversities, which is more detrimental to mental health than any single adversity, thereby significantly affecting the psychological and personality health of older adults.

### 4.3 Analysis of the moderating effect of social participation On childhood adversity and depression in older adults

Results of the moderating effect study suggest that social participation appears to reduce the negative impact of “poor family economic situation” on depression in early childhood environments for older adults of all ages. This is mainly because social participation in later life can benefit older adults and may compensate for a lack of social participation in their youth due to insufficient economic resources. In the meantime, various forms of social participation can enhance their sense of self-efficacy and social identity, weakening the inferiority complex resulting from low-income family economic standing in childhood. Additionally, it will increase the possibility of older adults getting social support, which will aid them in alleviating depression. For instance, community volunteer activities and interest groups can provide opportunities for older adults to interact and collaborate with others, thereby expanding their social support network and improving their mental health. Recreational activities such as dancing and fitness not only promote physical health but also enhance emotional and psychological well-being through social interaction.

However, social participation can exacerbate the problem of “a lack of mutual assistance within the neighborhood” and “a lack of good playmates” during the early stages of life. As a result of this counterintuitive finding, we can observe that social participation regulation is heterogeneous in response to different influences of childhood adversity and depression. According to studies, when designing the living environment for older adults with emotional disorders, it is essential to consider the emotional instability of the older adults due to illness so that an environment can be stable and familiar (Tu, 2016). Researchers will examine the environment’s ability to evoke positive emotions and strengthen inherent memories (Liu and Zhu, 2019). In addition, some studies have found that music can assist older adults with cognitive disorders by evoking their happy memories to prevent and degrade their agitated behavior (Pan, 2013). Memories and emotions are triggered by social environments and specific characteristics (Ellis et al., 2011), and similar environments evoke the same memories and emotions. Inferentially, childhood adversity, which occurred in the social environment in the early years, as well as “lack of mutual assistance within the neighborhood” and “lack of good playmates,” have profoundly affected children’s mental health. In their later years, older adults usually engage in social participation behaviors in their social environment. During this time, more social participation behaviors are likely to evoke negative memories of the trauma experienced in the social environment during the early years of older adults, which will further exacerbate their depression tendencies and damage their mental health. At the same time, social participation will aggravate the depression degree of “lonely due to lack of friends” in the early social environment for the oldest-old; the reasons are similar to the mechanism of the above two regulatory effects. Nevertheless, this adjustment has a significant effect only on depression among the oldest-old. There is a possibility that “lonely due to lack of friends” is more harmful to children than “lack of good playmates.” Compared to the young-old with the same childhood adversity, the oldest-old feel more loneliness due to lack of friends in the early years. Meanwhile, more of the oldest-old are experiencing physical and degenerative changes, and social participation will require more energy. In conjunction with the above factors, the adjustment of social participation worsens the psychological condition of the oldest-old who are “lonely due to lack of friends” during their early years. Due to their low psychological resilience, older adults who have experienced childhood adversity are more likely to suffer from depression if they participate excessively in social activities, so they should be encouraged to participate moderately in social activities.

In this study, there is strong evidence to suggest that social participation moderates the relationship between childhood adversity and depression in older adults, which suggests that social participation may not only cushion the impact of childhood adversity but may also regulate some of the influence between childhood adversity and depression. Therefore, this study proposes the following recommendations: Clinical interventions can guide older adults toward moderate social participation to enhance resilience and reduce depressive symptoms, especially for depression patients with a history of childhood adversity, by providing personalized treatment plans. Increasing moderate social participation enables older adults to access more social support and emotional interaction, thereby enhancing self-efficacy and psychological resilience, effectively alleviating symptoms of depression. Additionally, governments and communities should collaborate to create opportunities and platforms for older adults to engage in social activities. Tailored social participation activities should be designed specifically for older adults with different childhood adversities to avoid triggering negative memories. For example, organizing low-intensity, low-pressure social activities such as cultural and recreational events, craft workshops, etc., can help older adults build new positive memories and social connections, promoting psychological well-being. Furthermore, additional psychological protection measures should be provided for older adults reporting childhood adversity, such as establishing professional psychological counseling and support services, offering regular mental health assessments and interventions to help them manage the impact of early-life trauma. Through these preventive measures, the health risk chain from childhood to late adulthood can be broken, improving the psychological health status of older adults and reducing the likelihood of depression occurrence.

### 4.4 Limitation

There are some limitations to this study as well. Firstly, it is a cross-sectional study, which limits our ability to infer causal relationships regarding the long-term development of depression levels. Future research should employ longitudinal study designs to track changes in depressive symptoms among older adults over time, enabling a more robust establishment of causal relationships over time. At the same time, due to the research objectives, this study did not utilize the most recent 2020 CHARLS data. This choice may limit the timeliness and accuracy of the findings. Future research will consider using the latest data sets to enhance the comprehensiveness and precision of the study. Secondly, older adults may experience recall bias when recalling childhood adversity, potentially impacting the accuracy of study outcomes. Cognitive decline in older adults may hinder accurate recollection of childhood experiences, leading to memory omissions or errors. Such recall bias could either underestimate or overestimate the impact of childhood adversity on depression in older adults. To mitigate this issue, the CHARLS questionnaire was designed to be as detailed and specific as possible, aiming to minimize recall bias among respondents. However, this measure cannot completely eliminate the influence of bias. Therefore, future studies could consider using more objective measurement tools and methods, such as corroborating self-reports with family members’ recollections or community records. Thirdly, while this study’s sample represents the middle-aged and older adults in China, its findings may be influenced by cultural backgrounds. Future research should delve deeper into the cultural specificity of study results and consider conducting cross-cultural comparative studies to better understand the impact of childhood adversity on psychological health among older adults across different cultural contexts. Finally, although this study addressed selection bias through propensity score matching, endogeneity issues due to omitted variables may still exist given our focus on the impact of childhood adversity on current depressive symptoms in older adults. Future research should further explore and control for other potential factors that may influence both childhood adversity and depressive symptoms in later life to enhance the robustness and credibility of the results.

## Data availability statement

The datasets presented in this study can be found in online repositories. The names of the repository/repositories and accession number(s) can be found at: http://charls.pku.edu.cn/en.

## Ethics statement

The studies involving humans were approved by the Institutional Review Board at Peking University. The studies were conducted in accordance with the local legislation and institutional requirements. Written informed consent for participation was not required from the participants or the participants’ legal guardians/next of kin in accordance with the national legislation and institutional requirements.

## Author contributions

PW: Methodology, Project administration, Writing – review & editing. XC: Writing – original draft, Writing – review & editing, Conceptualization, Methodology, Software. NZ: Writing – original draft, Data curation, Formal analysis, Resources, Writing – review & editing. HL: Software, Writing – original draft.
